# American Dental Association

**Published:** 1881-09

**Authors:** 


### EDITORIAL.

### AMERICAN DENTAL ASSOCIATION.

The annual meeting of the American Dental Association was held in New York, July 12th to 15th; about one month earlier than usual, on account of the prospective absence of many of the permanent members, in August the usual time of the meeting. Under the circumstances the attendance was very good, about one hundred and twenty. There was necessarily a little diversion of attention, but notwithstanding, there was a good, interesting, and instructive meeting.

There seems to be on the part of the members a growing desire to make the Association more efficient than it has been. Some of the embarassments that have existed in the estimation of some, at least, have become much less, if not altogether removed. This body should have the best efforts of its members; such efforts should have in view the best interests of the body and of the profession, whose instrument it is. Several of the standing committees presented their reports; upon them much excellent discussion was had. Some other reports for want of time were not ready, which it is to be hoped will be in readiness for next year, when we hope there will be one of the best meetings of this body ever held; that is to held in Cincinnati at the usual time.

The officers for the present year are President, H. A. Smith, Cincinnati; First Vice-President, W. C. Barrett, Buffalo; Second Vice-President, G. J. Fredericks, New Orleans; Recording Secretary, G. H. Cushing, Chicago; Corresponding Secretary, A. M. Dudley, Salem, Mass; Trersurer, W. H. Goddard, Louisville, Ky; Chairman of the Executive Committee, J. N. Crouse, Chicago.

The transactions will appear at an early date, the perusal of which will be far more satisfactory than any partial reports that can be given in these pages.

